# Inhibitory effect of licorice extract on the germination and outgrowth of *Paraclostridium bifermentans* spores

**DOI:** 10.3389/fmicb.2022.1076144

**Published:** 2022-12-02

**Authors:** Mengmeng Song, Yang Lei, Ahtisham Ali, Yan Xu, Kairan Sheng, Tianran Huang, Jichao Huang, Ming Huang

**Affiliations:** ^1^Key Laboratory of Meat Processing and Quality Control, Ministry of Education, College of Food Science and Technology, Nanjing Agricultural University, Nanjing, China; ^2^Jiangsu Research Center for Livestock and Poultry Products Processing Engineering Technology, Nanjing Huangjiaoshou Food Science and Technology Co., Ltd., Nanjing, China; ^3^College of Engineering, Nanjing Agricultural University, Nanjing, China

**Keywords:** *Paraclostridium bifermentans*, spores, licorice extract, germination, outgrowth, inhibition

## Abstract

**Introduction:**

*Paraclostridium bifermentans* is responsible for spoilage properties in vacuum-packaged meat. Ordinary heat treatment techniques are ineffective to control the extremely heat-resistant spores of *P. bifermentans*. Therefore, finding a new strategy to prevent the contamination of *P. bifermentans* spores in vacuum-packaged meat is challenging.

**Methods:**

In this study, *P. bifermentans* was isolated from the vacuum-packaged chicken, and the inhibitory effects of licorice extract on the germination and outgrowth of *P. bifermentans* spores, as well as the key bioactive components in the licorice extract involved in inhibiting spore activity, were investigated.

**Results:**

The spores induced by combination-nutrient-germinant (150 mmol/L L-alanine and 20 mmol/L inosine, co-AI) did not germinate when the concentration of licorice extract was ≥ 3.13 mg/ml. The germination of *P. bifermentans* spores induced by non-nutrient-germinant (8 mmol/L dipicolinic acid, DPA) was completely prevented by licorice extract at least 1.56 mg/ml. While the outgrowth of *P. bifermentans* spores was inhibited at a concentration of 0.39 mg/ml. Licorice extract did not seem to damage the non-germinated spores but blocked the germinant sensing. Licorice extract prevented the outgrowing spores from becoming vegetable cells by disrupting the inner membrane. Furthermore, the results obtained from LC-MS data analysis exhibited 15 key bioactive compounds in licorice extract, such as glycyrrhizic acid, liquiritin, etc. Among them, glycyrrhizic acid and liquiritin apioside exerted efficient inhibitory properties on the germination and outgrowth of *P. bifermentans* spores.

**Discussion:**

This present study demonstrated that licorice extract can be used as a promising inhibitor of spores and provides a new method to control the residual *P. bifermentans* spores in meat products. Meanwhile, this study exhibits a baseline for the better understanding of the potential application of licorice extracts to control the *P. bifermentans* spores in meat products.

## Introduction

*Paraclostridium bifermentans* was first isolated from putrefied meat by Tissier and Martelly in 1902 (Sankar et al., 2018). Later, it was also discovered in cured meat, dry smoked sausages, and cooked meat products, respectively (Kokubo et al., 1986; Matos et al., 2006). *P. bifermentans* is a Gram-positive, rod-shaped, gas-producing, sulfite-reducing, endospore-forming anaerobe, and as a clostridia, it has been able to lead to the corruption of food (Kutsuna et al., 2019). Due to the anaerobic requirement of *P. bifermentans* vegetative cells, it has been able to survive in the form of dormant spores to resist heat, radiation, desiccation, toxic chemicals, or other environmental stress factors (Cui et al., 2011). In recent years, few research studies such as Bhattacharjee and Sorg (2018) have reported on *P. bifermentans* in meat. Furthermore, there are several reports have been found on its control strategy, especially with its heat-resistant spores. Therefore, this study has been conducted to monitor the controlling influence of licorice extracts to inhibit the activity of *P. bifermentans*.

Spore germination is a series of irreversible biophysical and biochemical reactions. Once the condition becomes conducive, the spores can be germinated and undergo outgrowth where they are finally converted to vegetative cells (Zhu et al., 2022). Setlow et al. (2017) conducted a systematic study of spores and summarized several elements regarding the reception of germination signal, the release of DPA, the hydrolysis of peptidoglycan cortex, and the resumption of metabolism, respectively. Notably, in contrast to the *Bacillales* spores, the DPA release of *Clostridiales* spores only occurs after cortex degradation.

Heat sterilization has been known as the most frequently-used technology in food-processing industries. However, ordinary heat treatment techniques are ineffective to control extremely heat-resistant spores (Zhu et al., 2022). According to previous studies, it has been investigated that enhancing the sterilization strength can inactivate spores in food but equally bring adverse impacts on product texture, flavor, and nutritional quality. Therefore, exploring an appropriate strategy that can substitute conventional sterilization methods to improve the palatability of processed foods and control the germination of spores has become a focus of research (Alnoman et al., 2015).

Since the majority of the resistance features of spores would disappear once germination starts, it is typically an efficient way to immediately inhibit the outgrowth of spores after the onset of germination. It is also feasible to directly prevent spore germination. Previous research has shown that chemical antimicrobials such as sorbate, benzoate (Alnoman et al., 2015), potassium lactate, and sodium diacetate (Redondo-Solano et al., 2021) showed inhibitory effect against the germination and outgrowth of spores. However, as consumers' attitudes toward chemical preservatives change, natural antimicrobials are more likely to be accepted (Juneja et al., 2007).

Natural plant extracts have been extensively studied as chemical antimicrobial substitutes due to their industrial advantages worldwide. They contain a high concentration of bioactive components, which help to maintain a good balance of food flavor and microbiological safety (Alanazi et al., 2018). Numerous studies have shown that green tea extracts (Friedman, 2007) and grape seed extract (Cosansu and Juneja, 2018) have inhibitory activities against various spore-forming bacteria. Licorice is a “homology of medicine and food” plant with ideal sweetness and excellent antibacterial properties. It has been classified as Generally Recognized as Safe (GRAS), representing that its addition to food in typical concentrations is permitted (Han et al., 2021). A variety of bioactive components contained in licorice are triterpenoids and flavonoids, which have attracted attention as excellent antibacterial agents. Most scholars have studied the inhibitory effect of licorice extracts on vegetable cells of spore-forming bacteria (Ambrico et al., 2020; Gholami et al., 2021). While there has been little research on the anti-spore capacity of licorice extracts, more research is needed.

**Figure 1:**
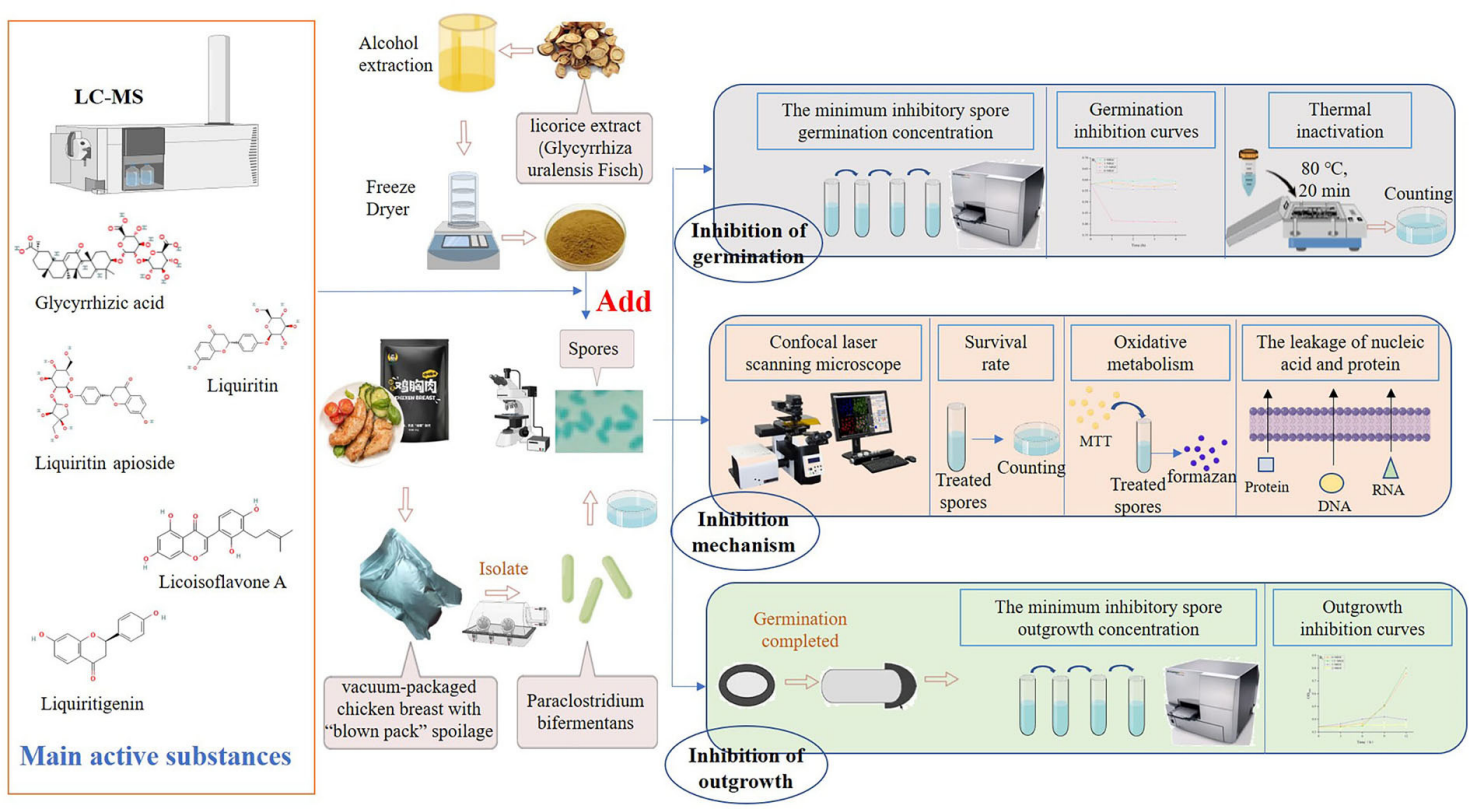
Design of experiments. The licorice extract was obtained from the root of licorice (*Glycyrrhiza uralensis* Fisch), and its main bioactive compounds were detected by LC-MS. *P. bifermentans* was isolated from the vacuum-packaged chicken breast with “blown pack” spoilage and cultured under anaerobic conditions to obtain the spores. The experiment was divided into two parts, on the one hand, the ability of licorice extract to directly inhibit the initiation of spore germination was determined, and on the other hand, the effect of licorice extract to prevent the outgrowth of germinated spores was investigated. Subsequently, the pattern of the germination and outgrowth inhibited by licorice extracts was explored. Finally, the contributions of the five bioactive compounds identified from licorice extracts in the inhibition of germination and outgrowth were measured.

The anti-germination and anti-outgrowth activities of licorice extract were judged by the minimum inhibitory spore germination concentration (MIGC) and the minimum inhibitory spore outgrowth concentration (MIOC), respectively. The mechanisms for the inhibition of licorice extract on spores were evaluated by confocal laser scanning microscopy (CLSM), detection of formazan, leakage of contents, and calculation of survivor rate. Finally, the main bioactive substances in licorice extract were identified by LC-MS and their contribution to the inhibition of spore germination and outgrowth was examined (Figure 1). The objective of this present study was to examine the inhibitory effects of licorice extracts on the germination and outgrowth of *P. bifermentans* spores. This study will provide a theoretical basis for controlling the *P. bifermentans* spores and avoiding contamination from spores in meat.

## Materials and methods

### Chemicals and bacterial culture

Licorice root (*Glycyrrhiza uralensis Fisch*) was obtained from Yonggang Co., Ltd (Haozhou, China). Reinforced clostridium medium (RCM, 10 g tryptone, 10 g beef powder, 3 g yeast powder, 5 g glucose, 1 g soluble starch, 5 g NaCl, 3 g CH_3_COONa, 0.5 g L-cysteine hydrochloride, 0.5 g agar powder, and pH 6.8), reinforced clostridium agar (RCA, 10 g tryptone, 10 g beef powder, 3 g yeast powder, 5 g glucose, 1 g soluble starch, 5 g NaCl, 3 g CH_3_COONa, 0.5 g L-cysteine hydrochloride, 12.5 g agar powder, and pH 6.8), spore staining solution, D-glucose, D-fructose, D-galactose, D-xylose, L-alanine, L-aspartate, L-valine, L-proline, L-histidine, L-leucine, inosine, dipicolinic acid (DPA), and rutin were purchased from HaiBo Co., Ltd (Qingdao, China). Thiazolyl blue tetrazolium (MTT), glycyrrhizic acid, liquiritigenin, and dimethyl sulfoxide were purchased from Solarbio Science & Technology Co., Ltd (Beijing, China). Liquiritin, liquiritin apioside and licoisoflavone A were purchased from Yuan-Ye Bio-Technology Co., Ltd (Shanghai, China). Gallic acid was from Macklin Biochemical Co., Ltd (Tianjin, China). All the chemicals used in this study were of analytical grade.

*P. bifermentans* was isolated from the vacuum-packaged chicken breast with “blown pack” spoilage obtained from Nanjing Huangjiaoshou Food Science and Technology Co., Ltd. Nanjing, China. The isolation process strictly followed the operation process and the bacteria have been identified by 16S rRNA sequence analysis and uploaded to the NCBI database to get the serial number: ON078500. The strain was inoculated into RCM and incubated overnight in an anoxia workstation (Bugbox M, Ruskinn, Britain) at 37°C. The system was regulated by three gases: N_2_, a mixture of 5% H_2_ and N_2_, and CO_2_, where the CO_2_ content was set at 5%. Then, the culture broths were transferred to RCM broth containing 25% glycerol and stored at −80°C until used (Bhattacharjee and Sorg, 2018).

### Spore preparation and purification

Spore suspensions of *P. bifermentans* were prepared and purified as described previously by Alnoman et al. (2017). Briefly, 100 ml activated cultures of *P. bifermentans* were streaked onto Duncan–Strong Sporulation Media (DSSM) (Duncan and Strong, 1968) agar plates (15 g tryptone, 4 g yeast powder, 10 g Na_2_HPO_4_, 4 g D-raffinose pentahydrate, 1 g sodium thioglycolate, 20 g agar powder, 1,000 ml pure water, and pH 7.5) and incubated anaerobically at 37°C for 7 days. The cultures were scrapped into cold sterile water (4°C) and washed 3–4 times. Harvested spores were then preserved at 4°C for 24 h and heat-shocked at 80°C for 20 min to inactivate the remaining vegetative cells. It can be observed that the spores were stained greenish-blue after spore staining (spore staining solution), and the spore suspensions were > 99% pure when observed under the upright fluorescence microscope (Scope.A1, Carl Zeiss, Oberkochen, Germany). The OD_600_ of purified spores was adjusted to 1.0.

### Preparation of licorice extract

The ethanol-soluble extract of licorice was prepared as previously described by Cui et al. (2011). The licorice powder was socked in 75% ethanol and extracted by ultrasound-assisted methods and freeze-dried to obtain the licorice extract.

### The determination of the total content of flavonoids, polyphenols, and DPPH free radical scavenging rate

The total flavonoid compound (TFC) content of licorice extract was measured following the aluminum chloride colorimetric method (Sheng et al., 2013) with slight modifications. Approximately 500 ml of diluted solution of licorice extract was mixed with 70 ml of 5% (w/w) NaNO_2_, maintained for 5 min, and then 150 ml of 10% (w/w) AlCl_3_ was added to the mixture. After 5 min, 500 ml of 1 mol/L NaOH and 1,300 ml pure water were added. The absorbance of the mixture was measured at 415 nm. The calibration curve (y = 0.006x + 0.0585, where y is the absorbance value of the sample, and x is sample concentration) ranged from 0 to 60 μg/ml (R^2^ = 0.9991). The total polyphenolic compound (TPC) content was determined by using the Folin–Ciocalteu method (Yu et al., 2017) with some modifications. A mixture of the 1-ml diluted solution of licorice extract and 5 ml of 10% (w/w) Folin–Ciocalteu reagent was prepared, and 4 ml of 7.5% (w/w) sodium carbonate solution was added into a solution after 5 min. Then, the mixture was left to stand in the dark for 1 h, and absorbance was measured at 765 nm. The calibration curve (y = 0.0012x + 0.0697, where y is the absorbance value of the sample, and x is the sample concentration) ranged from 0 to 80 μg/ml (R^2^ = 0.9991). The contents of flavonoids and polyphenols were expressed as mg/g equivalent to rutin and gallic acid. The determination of the scavenging effect on the DPPH radical was based on the method of Shi et al. (2021) and some modifications were made. The serial dilutions of licorice extract were mixed with an equal amount of DPPH radical dot (0.2 mmol/L, prepared in anhydrous alcohol) and maintained in the dark for 30 min, the absorbance value was measured at 517 nm. The DPPH radical dot clearance rate (%) was calculated by using the following equation:


(1) $$\begin{array}{l} \text{DPPH radical dot clearance rate }\left(\%\right)\\ \;=\;\frac{\left(C_{blank}-C_{sample}\right)}{C_{sample}}\;\times 100\% \end{array}$$

C_blank_: OD_517_ of the control reaction; C_sample_: OD_517_ of the examined samples.

### Analysis of bioactive components in licorice extract by LC-MS

The samples were analyzed by LC-MS system (TripleTOF 5600+, AB Sciex Instruments), and data acquisition and processing were performed using PickView 2.0. A 10-ml solution was injected into the LC column (Kinetex C_18_ 2.6 μm 100 × 2.1 mm) with a flow rate of 0.3 ml/min. The column temperature was 4 °C. Buffer A consisted of 0.1% formic acid in the water, and Buffer B was methanol. The gradient was 5% Buffer B for 1 min, 5–95% Buffer B for 14 min, and 95% Buffer B for 3 min.

The sample was analyzed by mass spectrometer with direct infusion of the sample into the electrospray ionization source operating in the negative ion mode. Curtain gas, ion source gas and auxiliary gas were 40 psi, 65 psi and 65 psi, respectively. IonSpray voltage floating, collision energy and declustering potential were set to −4500 V, −10 V and −80 V, respectively, with a gasification temperature of 550 °C.

### Inhibitory effect of licorice extract on the germination of *P. bifermentans* spores

#### Determination of spore germination

Spores can be observed to change from phase-bright to dark phase during germination, and the optical density at 600 nm (OD_600_) of the spores will drop by 60% when the spores germinate completely (Zhu et al., 2020). Therefore, the germination rate can be calculated through the change in absorbance value. The licorice extract was added into a sterile 25 mmol/L Tris-HCl buffer (pH = 7.4) containing DPA or co-AI. DPA and co-AI were two selected germinants with well-germination-promoting effects (Supplementary Figure S1, Supplementary Tables S1, S2), respectively. The concentration range of licorice extracts has achieved the desired effect after pre-experiments. Therefore, the licorice solution was subjected to a two-fold serial dilution with final concentrations ranging from 100 to 0.20 mg/ml (100, 50, 25, 12.5, 6.25, 3.13, 1.56, 0.78, 0.39, and 0.20 mg/ml). *P. bifermentans* spore suspensions were heat-activated at 80 °C for 15 min and cooled in cold water for 5 min prior to mixing with an equal amount of the licorice solutions and cultivated in an anoxia workstation at 37°C for 1 h (Chaibi et al., 1997). Then, the OD_600_ of treated spores was monitored using an enzyme-labeled instrument (M2e, MD, America). The germination inhibition rate was measured by Alanazi et al. (2018). The germination inhibition rate (%) can be calculated as follows:


(2) $$\begin{array}{l} \text{Germination inhibition rate }\left(\text{\%}\right)=100-\frac{B_{T}}{B_{C}}\times 100\% \end{array}$$

B_*T*_: decrease of OD_600_ in treatment; B_C_: decrease of OD_600_ in control.

The minimum concentration that completely inhibited the germination of *P. bifermentans* spores was MIGC.

Based on MIGC, the change of OD_600_ was measured at predetermined intervals from 0–4 h when treated with licorice extract (1 × MIGC, 2 × MIGC, 1/2 × MIGC) and incubated anaerobically at 37°C, to obtain the germination curve. The control group was Tris-HCl buffer containing DPA or co-AI with no licorice extract.

#### Further verification of spore germination

The germinated spores were killed by heat treatment since the resistance of spores would decrease after germination, while the non-germinated spores survived (Cui et al., 2011). Spore suspensions treated with licorice extract were exposed to a water bath at 80°C for 20 min and cooled immediately. Subsequently, 100 ml diluents with appropriate concentrations were inoculated onto RCA under anaerobic conditions at 37°C for 24 h, and the survivor enumerations were conducted. The levels of licorice extract carried from Tris-HCl into RCA had no effect.

### Inhibitory effect of licorice extract on the outgrowth of *P. bifermentans* spores

Heat-activated spore suspensions of *P. bifermentans* were suspended in 25 mmol/L Tris-HCl buffer (pH = 7.4) containing DPA or co-AI and incubated anaerobically at 37°C for 1 h to reach the maximal extent of germination (Chaibi et al., 1997). Subsequently, the spores were transferred into RCM containing various concentrations of licorice extract (0, 0.20, 0.39, 0.78, 1.56, and 3.13 mg/ml) and incubated anaerobically at 37°C for 12 h. According to the pre-experiment, the outgrowing spores grew rapidly in RCM within 12 h. Therefore, it is considered that the outgrowth of *P. bifermentans* spores was blocked if the increase of OD_600_ was <0.1 after 12 h, and the minimal concentration of licorice extract at this point was the MIOC.

Based on MIOC, the OD_600_ of spores that incubated anaerobically in RCM containing licorice extract (1 × MIOC, 2 × MIOC, and 1/2 × MIOC) was measured at different periods (0, 3, 6, 9, and 12 h) to obtain the outgrowth curves. The control group was RCM without licorice extract.

### Confocal laser scanning microscope (CLSM) analysis

Germination-inhibited spores (treated by licorice extract at MIGC) and outgrowing spores (treated by licorice extract at MIOC) were fixed on sterile slides, stained with the membrane-impermeable stain PI (3 μl in 1 ml), and kept in the dark at room temperature for 45 min (Pappas et al., 2015). Fluorescence was observed by CLSM (63 × oil mirror) at an excitation wavelength of 490 nm and an emission wavelength of 635 nm (Sunny-Roberts and Knorr, 2008).

### Survival rate of *P. bifermentans* spores

Treated spores were diluted to the appropriate concentration and cultured in RCA medium under anaerobic conditions at 37 °C for 24 h. The total number of colonies was counted. The levels of licorice extract carried from Tris-HCl or RCM into RCA had no effect.

### Determination of oxidative metabolism

The previous experiments showed that the licorice extract did not seem to damage the non-germinated spores, but had an inactivating effect on the outgrowing spores. Therefore, the subsequent experiments targeted the outgrowing spores. MTT colorimetric method is a frequently used method for evaluating metabolic activity based on the reduction of tetrazolium salt to purple formazan by oxidative metabolism, and the process of oxidative metabolism was assessed by detecting the content of formazan (Oh and Hong, 2021). Approximately, 200 ml of treated spores were taken at selected periods and 50 ml of 5 mg/ml MTT was added. The mixtures were maintained at 37°C for 4 h in the dark and the supernatant was removed and replaced by dimethyl sulfoxide (DMSO). The content of formazan was measured at 570 nm.

### The leakage of nucleic acid and protein

Treated spores were immediately filtered (0.22 μm pore size), and the absorbance of the filtrate at 260 and 280 nm was measured to determine the amount of nucleic acid released (Cai et al., 2019).

### Inhibitory effect of bioactive components in licorice extract on the germination and outgrowth of *P. bifermentans* spores

A higher response value may represent a higher relative content. Meanwhile, their antibacterial activity as reported in the past literature (Quesada et al., 2012; Kong et al., 2015; Chakotiya et al., 2016), and their availability were taken into account. On this basis, glycyrrhizic acid, liquiritin, liquiritin apioside, licoisoflavone A, and liquiritigenin were selected for further validation experiments. The bioactive components prepared in DMSO were diluted 1/1,000 in RCM or 25 mmol/L Tris-HCl (pH = 7.4, containing germinants) buffer to 2 mg/ml, and the inhibitory solutions were diluted according to the two-fold serial dilution method. Their anti-germination and anti-outgrowth capacities to *P. bifermentans* spores were evaluated according to the method in 2.6.1 and 2.7.

### Statistical analysis

All data reported are averages of triplicate experiments. Data were imported to the Statistical Analysis System (SAS, Release 8.1; SAS Institute Inc., Cary, NC, USA) for the analysis of variance procedure (ANOVA) and Duncan's multiple range tests to obtain significant differences (*p* < 0.05) among treatments.

## Results and discussion

### Analysis of bioactive components in licorice extract by LC-MS

The yield of licorice extract was 12.8%. The content of total polyphenols of licorice extract was measured as 31.4 ± 0.46 mg GAE/g DW and the content of total flavonoids was 162.3 ± 9.18 mg RE/g DW. The DPPH radical dot clearance rate of licorice extract was positively related to its concentration (Supplementary Figure 2).

It is universally acknowledged that the anti-spore properties of natural plant extracts are related to the bioactive compounds (Alanazi et al., 2018). Likewise, the anti-spore function of licorice extract was derived from the bioactive components such as glycyrrhizic acid and flavonoids in licorice (Sharifi-Rad et al., 2021). In this study, the bioactive components of the prepared licorice extract (10 ppm, dissolve with 80% methanol) were analyzed by LC-MS (Table 1). The analysis of the data revealed the presence of 15 main bioactive components. Among them, glycyrrhizic acid, liquiritin, liquiritin apioside, licoisoflavone A, and liquiritigenin showed relatively high response values, suggesting that the content of these compounds in licorice extract may be relatively high. Zhang et al. (2022) discovered that licoflavonol, licoricone, quercetin, and licochalcone A were the main compositions in licorice. Quintana et al. (2019) proved that the most abundant compounds in the licorice extract were liquiritin, liquiritigenin, isoliquiritigenin, and glabridin. Possible reasons for the discrepancy are attributed to the quite variable composition and content of licorice extract as well as the difference in variety, origin, and methods of extraction.

**Table 1:** The main bioactive components in 75% alcohol extract of the licorice extract.

**Serial number**	**Inferred composition**	**Retention time (min)**	**Mass-to-charge ratio (M/Z)**	**Molecular formula**	**Intensity**	**Structural formula**
1	Glycyrrhizic acid	13.43	821.40	C_42_H_62_O_16_	107,685	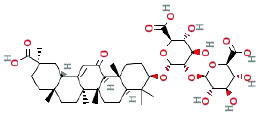
2	Liquiritin	7.81	417.12	C_21_H_22_O_9_	96,151	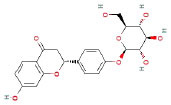
3	Liquiritin apioside	7.94	549.16	C_26_H_30_O_13_	65,944	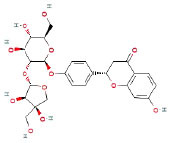
4	Licoisoflavone A	12.72	354.10	C_20_H_18_O_6_	48,265	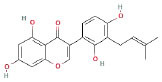
5	Liquiritigenin	9.4	255.07	C_15_H_12_O_4_	32,158	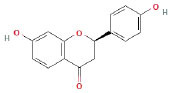
6	Glycycoumarin	12.96	368.12	C_21_H_20_O_6_	24,183	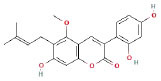
7	Isoangustone A	14.73	421.17	C_25_H_26_O_6_	22,177	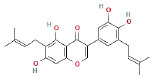
8	Licoisoflavone B	13.5	351.09	C_20_H_16_O_6_	21,674	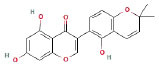
9	Licochalcone D	12.72	353.14	C_21_H_22_O_5_	4,856	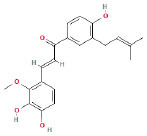
10	Formononetin	11.54	257.07	C_16_H_12_O_4_	4,817	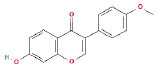
11	Licoflavone C	13.70	337.11	C_20_H_18_O_5_	3,865	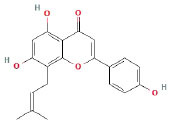
12	Licoricone	13.28	381.13	C_22_H_22_O_6_	2,182	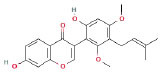
13	Barpisoflavone A	9.96	299.06	C_16_H_12_O_6_	1,893	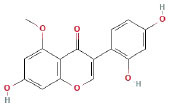
14	Naringenin	10.19	271.06	C_15_H_12_O_5_	1,807	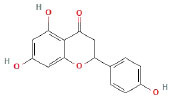
15	2'-O-Methylisoliquiritigenin	10.71	269.08	C_16_H_14_O_4_	1,783	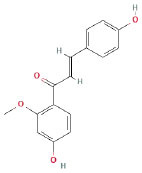

### Inhibitory effect of licorice extract on the germination of P. *bifermentans* spores

**Figure 2:**
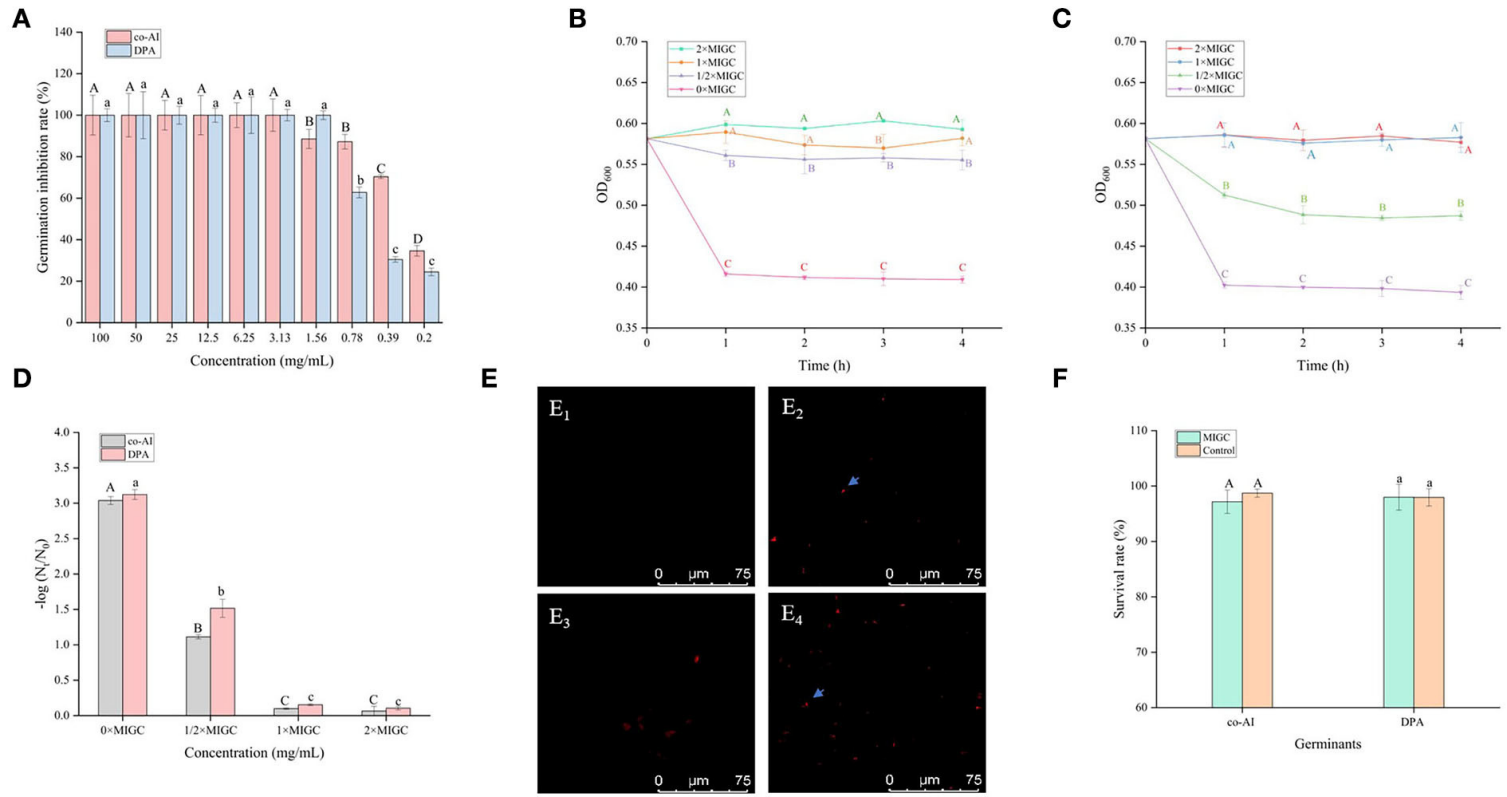
Inhibitory effect of licorice extract on the germination of *P. bifermentans* spores. **(A)** Inhibition of licorice extract on the germination of *P. bifermentans* spores in the presence of the combination-nutrient-germinant (150 mmol/L L-alanine and 20 mmol/L inosine, co-AI) and non-nutrient-germinant (8 mmol/L dipicolinic acid, DPA); Germination curves of *P. bifermentans* spores when treated with licorice extract at the minimum inhibitory spore germination concentration (MIGC, 3.13 mg/ml), 2 × MIGC (6.25 mg/ml), 1/2 × MIGC (1.56 mg/ml), and 0 × MIGC (0 mg/ml) in the presence of co-AI **(B)** and at 1 × MIGC (1.56 mg/ml), 2 × MIGC (3.13 mg/ml), 1/2 × MIGC (0.78 mg/ml), and 0 × MIGC (0 mg/ml) in the presence of DPA **(C)**; **(D)** Effect of licorice extract on the thermal inactivation of *P. bifermentans* spores; PI staining results of *P. bifermentans* spores when treated with licorice extract at 0 mg/ml **(E**_1_**)**, 3.13 mg/ml **(E**_2_**)** in the presence of co-AI and at 0 mg/ml **(E**_3_**)**, 3.13 mg/ml **(E**_4_**)** in the presence of DPA; **(F)** The survival rate of *P. bifermentans* spores after treatment with licorice extract at corresponding MIGC in the presence of co-AI or DPA. Values with different uppercase letters were significantly different (*p* < 0.05) in the co-AI-induced group and values with different lowercase letters were significantly different (*p* < 0.05) in the DPA-induced group **(A,D,F)**. Values with different uppercase letters were significantly different (*p* < 0.05) at the same time point in different concentration groups **(B,C)**. The error bars represent standard deviations of the mean (*n* = 3).

As shown in Figure 2A, all of the concentrations of licorice extract weakened the induced germination of *P. bifermentans* spores. The spores induced by co-AI or DPA did not germinate at a concentration of at least 3.13 or 1.56 mg/ml, respectively.

*P. bifermentans* spores germinated rapidly when induced by germinants within 1 h of anaerobic incubation at 37°C (Figures 2B,C). The germination of *P. bifermentans* spores treated with licorice extract (1 × MIGC, 3.13 mg/ml and 2 × MIGC, 6.25 mg/ml) did not occur in the presence of co-AI, and the blocking effect was continued up to 4 h or longer (Figure 2B). The DPA-induced spores did not germinate when treated with licorice extract (1 × MIGC, 1.56 mg/ml and 2 × MIGC, 3.13 mg/ml) (Figure 2C). These results suggested that the higher concentrations of plant extracts were devoted to prolonged inhibition of spore germination. Similarly, Alanazi et al. (2018), showed that higher concentrations (0.1%) of eugeno and carvacro could inhibit *Clostridium perfringens spore* germination for at least 1 h. However, the spores (co-AI-induced germination) treated with licorice extract (1/2 × MIGC, 1.56 mg/ml and 0 × MIGC, 0 mg/ml) germinated in the first 1 h, and the decrease in their absorbance values was significantly (*p* < 0.05) different from the remaining two groups (Figure 2B). When induced by DPA, licorice extract (1/2 × MIGC, 0.78 mg/ml) only had moderate inhibitory effects against spores and the OD_600_ at each time point was significantly (*p* < 0.05) lower than 1 × MIGC and 2 × MIGC groups (Figure 2C). Collectively, the lower concentration of licorice extract could not provide a well germination-inhibitory effect on *P. bifermentans* spores. A study demonstrated that the 0.05% chitosan exhibited less inhibitory effect than 0.1% (Alnoman et al., 2017).

The results of the thermal inactivation after heat treatment of the spores showed the same trend (Figure 2D). In the presence of co-AI, the thermal inactivation of spores treated with licorice extract (1 × MIGC, 3.13 mg/ml and 2 × MIGC, 6.25 mg/ml) was significantly (*p* < 0.05) lower than that of groups containing ½ × MIGC (1.56 mg/mL) and 0 × MIGC (0 mg/ml) licorice extract. In the presence of DPA, few heat-inactivated spores were found in the high-concentration licorice (1 × MIGC, 1.56 mg/ml and 2 × MIGC, 3.13 mg/ml) treatment group.

Therefore, these results confirmed the role of licorice extract in intense inhibition activity against the germination of *P. bifermentans* spores. This experiment found that the alcoholic extract of licorice was rich in polyphenols and flavonoids. Therefore, it can be speculated that their presence of them conferred a perfect germination inhibition effect on the licorice extract. The addition of 0.5–2% (w/w) green tea extract (with high-level catechins) reduced the potential harm caused by *C. perfringens* spore *via* germination (Juneja et al., 2007). The same result confirmed that grape seed extract considered a rich source of polyphenols and flavonoids inhibited the germination of *Alicyclobacillus acidoterrestris* spores in apple juice (Molva and Baysal, 2015; Zhao et al., 2021). In addition, the germination of *Clostridium botulinum* was arrested by essential oils of eucalyptus, chamomile, and cedar at a concentration of at least 300 ppm (equivalent to 0.3 mg/ml) (Chaibi et al., 1997). The lower concentration requirements suggest that the ability to inhibit spore germination might be not only involved in extracts but also related to the difference in strains as well as the participation of germinants.

As shown in Table 2, it could be discovered that the anti-germination effects of these bioactive substances were in a dose-dependent manner. Glycyrrhizic acid and liquiritin apioside with relatively high concentrations expressed strong anti-germination effects. Evidently, the MIGC of glycyrrhizic acid was 2 and 1 mg/ml when the spores were triggered by co-AI and DPA, separately, and their inhibitory effects were significantly (*p* < 0.05) different from the remaining concentrations. Liquiritin apioside at the concentration of 2 mg/ml completely blocked the co-AI-induced germination, and the inhibition rate was significantly (*p* < 0.05) higher than that of the diluted concentration. Meanwhile, only slight germination appeared in DPA-induced spores when treated with 2 mg/ml liquiritin apioside. Attentively, as the concentrations were diluted, they retained a well-inhibitory effect that the germination inhibition rate of them was at least 50%. In the presence of co-AI, liquiritin (2 and 1 mg/ml) inhibited nearly 60% of germination, while liquiritigenin and licoisoflavone A possessed similar anti-germination properties that they all exerted some degree of the inhibitory effect (22.71–38.79%). In the presence of DPA, liquiritin followed liquiritin apioside in its anti-germination property, which was able to enhance the germination inhibition rate to 57.79% at the concentration of 2 mg/ml; licoisoflavone A and liquiritigenin presented an inhibition rate of 19.47–37.74%.

**Table 2:** Inhibitory effect of bioactive compounds in licorice extract on the germination of *P. bifermentans* spores.

**Germinant**	**Measurement index**	**Concentration (mg/mL)**	**Bioactive compound**				
			**Glycyrrhizic acid**	**Liquiritin**	**Liquiritin apioside**	**Licoisoflavone A**	**Liquiritigenin**
co-AI	Germination inhibition rate (%)	2	104.39 ± 0.46^A^	59.24 ± 2.81^A^	105.70 ± 0.49^A^	38.79 ± 2.37^A^	35.08 ± 1.98^A^
		1	94.94 ± 0.32^B^	57.98 ± 1.42^A^	71.06 ± 3.04^B^	23.51 ± 1.41^B^	33.34 ± 2.44^AB^
		0.5	87.98 ± 0.30^C^	29.74 ± 2.4^B^	68.46 ± 2.72^B^	23.30 ± 1.50^B^	30.79 ± 2.36^B^
		0.25	76.61 ± 3.31^D^	29.27 ± 1.15^B^	57.39 ± 1.15^C^	21.55 ± 1.58^BC^	29.45 ± 2.62^B^
		0.13	73.28 ± 1.89^E^	20.59 ± 1.71^C^	51.48 ± 4.34^D^	18.74 ± 0.70^C^	22.71 ± 1.30^C^
DPA	Germination inhibition rate (%)	2	115.11 ± 0.71^A^	57.79 ± 2.92^A^	87.86 ± 7.52^A^	35.84 ± 3.10^A^	37.74 ± 3.11^A^
		1	109.87 ± 0.64^B^	55.42 ± 5.02^A^	79.78 ± 7.47^A^	30.54 ± 3.03^B^	30.01 ± 0.33^B^
		0.5	90.63 ± 1.78^C^	46.50 ± 4.16^B^	63.69 ± 3.09^B^	25.99 ± 2.07^C^	21.62 ± 1.37^C^
		0.25	78.02 ± 1.89^D^	45.13 ± 3.53^B^	63.45 ± 3.86^B^	25.76 ± 1.13^C^	21.93 ± 1.73^C^
		0.13	73.51 ± 3.11^E^	40.29 ± 2.47^B^	62.29 ± 4.12^B^	23.05 ± 1.39^C^	19.47 ± 1.59^C^

Heat-activated spores of P. bifermentans were mixed with the five compounds at various concentrations in the presence of the combination-nutrient-germinant (150 mmol/L L-alanine and 20 mmol/L inosine, co-AI) and non-nutrient-germinant (8 mmol/L dipicolinic acid, DPA) and incubated anaerobically at 37 °C for 1 h to obtain the germination inhibition rate of each compound. Results are averages of 3 trials and expressed as the means ± standard error. Values with different uppercase letters were significantly different (p < 0.05) in different concentrations.

**Figure 3:**
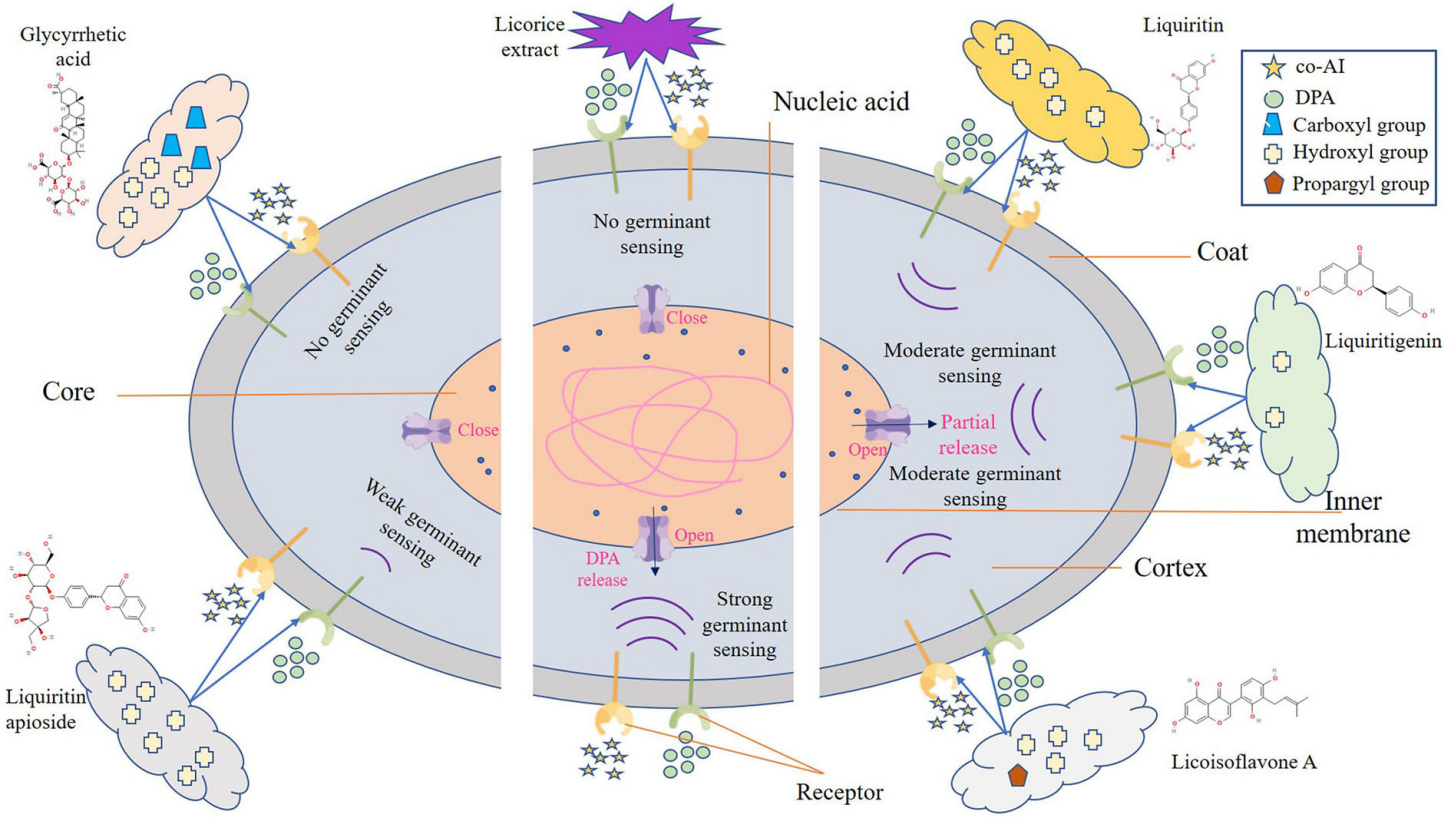
Schematic model of the *P. bifermentans* spore germination inhibited by licorice extract. Germinants (co-AI, 150 mmol/L L-alanine, and 20 mmol/L inosine; DPA, 8 mmol/Ldipicolinic acid) are sensed by interactions with germination receptors. Subsequently, the germination of spores begins and is accompanied by the hydrolysis of the cortex and leakage of DPA. However, the binding of the germinants to the receptor was affected by licorice extract, resulting in the no occurrence of germinant sensing. Similar situations happen to glycyrrhizic acid and liquiritin apioside, which contain more hydroxyl or carboxyl group, expanding their ability to prevent the reaction between the germinants and the receptors. As to liquiritin, licoisoflavone A, and liquiritigenin have relatively few hydroxyl groups, they are unable to completely inhibit the germinant sensing, and the spores will germinate partially.

One possible reason for the germination inhibition of licorice extract is the inactivation of spores by disruption of its structure, another potential reason is related to blocking the response between the germinants and the receptors (Cortezzo et al., 2004; Cai et al., 2019). The spores with compromised membranes were stained by propidium iodide (PI) selectively and assessed accurately through the red fluorescence observed by CLSM (Kuyukina et al., 2014). For germination-inhibited groups, spores with red fluorescence were rarely observed as shown in Figures 2E_2_,E_4_. Similarly, no such spores were displayed in the control group (Figures 2E_1_,E_3_), suggesting that no PI has entered into the non-germinated spores. To confirm whether the licorice extract can inactivate the spores, the spores were subjected to survivor enumerations in RCA. As shown in Figure 2F, the survival rate of germination-inhibited spores was not significantly different from that of the control group, suggesting that no damage to non-germinated spores can be made by licorice extract, which was in line with the previous finding on *C. botulinum* spores (Cui et al., 2011). These results were probably related to the inability of licorice extract to disrupt the structural integrity of the non-germinated spores. As shown in Figure 3, the protective effect provided by the spore out layer (cortex and coat) with hard texture enhances the difficulty for licorice extract to damage the structural integrity of the non-germinated spores directly (Gut et al., 2011). Therefore, it was conjectured that licorice extracts may inhibit germination by preventing the interaction between co-AI or DPA and their receptors, like other inhibitors (Cortezzo et al., 2004). Previous research showed that inhibitors had rather different effects on the germination of *Bacillus subtilis* spores with nutrients or non-nutrients, which may account for the difference in the MIGC of licorice extract required to inhibit co-AI and DPA-induced germination in our experiments (Cortezzo et al., 2004). Bhattacharjee and Sorg (2018) hypothesized that the germination receptor of *P. bifermentans* spores was in the outer layer, based on the germination pattern of *Clostridium difficile*. Therefore, it can be speculated that the lack of protection provided by the spore coat makes licorice extracts more accessible to the germination receptor, bringing an apparent germination inhibition response. Generally, co-AI or DPA are sensed by interactions with germination receptors and the commitment to germinate follows the germinant sensing. Subsequently, the cortex hydrolysis, as well as DPA release, causes the germination of spores. However, in the presence of licorice extract, the interaction of co-AI or DPA with germination receptors is blocked, this process may be due to competitive effects, or attributed to the loss of combining capabilities of them, resulting in the no occurrence of germinant sensing (Setlow et al., 2017). Similarly, the inhibitory effect of the five bioactive substances on germination may also be attributed to the blocking effect, as previously described. Their differences in germination inhibition effect may be determined by the molecular structure to a certain extent, especially the substituent groups and the relative positions of them (Kong et al., 2015). Glycyrrhizic acid, a natural triterpene glycoside in licorice root, consists of glucuronic acid and glycyrrhetic acid, therefore, it is rich in carboxyl and hydroxyl groups, and provided with the surfactant properties that increase membrane permeability, which may be one of the reasons for its excellent germination-resistant characteristic (Qiu et al., 2019). Therefore, in this study, glycyrrhizic acid could completely prevent the generation of germinate sensing, resulting in the no occurrence of germination. It is generally considered that the presence of hydroxyls leads to the enhancement of the target binding ability of compounds in plant extract (Alcaráz et al., 2000). More hydroxyl groups are essential to hinder the binding of the germinants to the receptors. The parent compound (C6-C3-C6) of flavonoids is two benzene rings connected by a heterocyclic pyrane ring, and the hydroxyl group or the glycosidic group, which is attached to the parent, is the difference between liquiritigenin and liquiritin (Kong et al., 2015). The presence of four free hydroxyls in the glucoside group of liquiritin played an essential role in the anti-germination effect. Therefore, they both showed moderate levels of inhibition in germinate sensing, and liquiritin showed better inhibition than liquiritigenin. Liquiritin apioside has two additional hydroxyls than liquiritin, which account for its good anti-germination effect and help it in suppressing germination well. When the concentration of liquiritin apioside was appropriate, germinate sensing of spores basically does not occur, resulting in the closure of DPA channel and not proceeding of germination. While compared to liquiritigenin, licoisoflavone A contains two additional hydroxyl groups and one propargyl group. However, there is no significant improvement in its anti-germination activity, possibly due to the relative position of the hydroxyl group and the presence of the propargyl group. Therefore, there was still partial germinate sensing as well as the DPA release it brought.

### Inhibitory effect of licorice extract on the outgrowth of *P. bifermentans* spores

**Figure 4:**
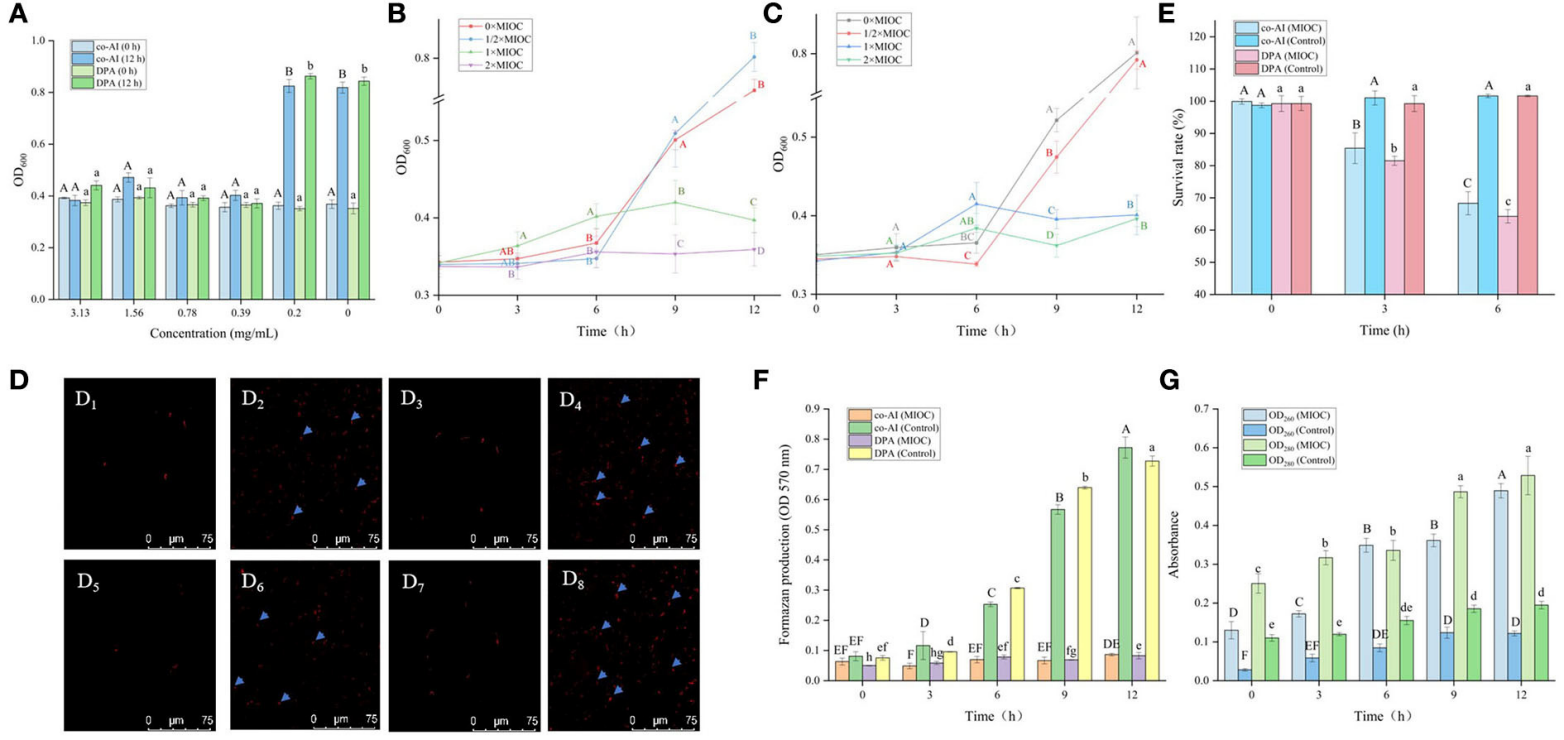
Inhibitory effect of licorice extract on the outgrowth of *P. bifermentans* spores. **(A)** Inhibitory of licorice extract on the outgrowth of *P. bifermentans* spores induced by the combination-nutrient-germinant (150 mmol/L L-alanine and 20 mmol/L inosine, co-AI) and non-nutrient-germinant (8 mmol/L dipicolinic acid, DPA); Outgrowth curves of *P. bifermentans* spores induced by co-AI **(B)** or DPA **(C)** when treated with licorice extract at the minimum inhibitory spore outgrowth concentration (MIOC, 0.39 mg/ml), 2 × MIGC (0.78 mg/ml), 1/2 × MIGC (0.19 mg/ml), and 0 × MIGC (0 mg/ml); PI staining results of *P. bifermentans* spores induced by co-AI when treated with licorice extract at 0 mg/ml **(D**_1_**)**, 0.39 mg/ml **(D**_2_**)** for 3 h and at 0 mg/ml **(D**_3_**)**, 0.39 mg/ml **(D**_4_**)** for 6 h or induced by DPA when treated with licorice extract at 0 mg/ml **(D**_5_**)**, 0.39 mg/ml **(D**_6_**)** for 3 h and at 0 mg/ml **(D**_7_**)**, 0.39 mg/ml **(D**_8_**)** for 6 h; **(E)** The survival rate of *P. bifermentans* spores induced by co-AI or DPA when treated with licorice extract at MIOC for 3 h and 6 h; **(F)** Effects of licorice extract on the oxidative metabolism of *P. bifermentans* spores induced by co-AI or DPA; **(G)** Leakage of DNA and RNA from *P. bifermentans* spores induced by co-AI or DPA when treated with licorice extract at MIOC. Values with different uppercase letters were significantly different (*p* < 0.05) in the co-AI-induced group and values with different lowercase letters were significantly different (*p* < 0.05) in the DPA-induced group **(A,E,F)**. Values with different uppercase letters were significantly different (*p* < 0.05) at the same time point in different concentration groups **(B,C)**. Values with different uppercase letters were significantly different (*p* < 0.05) in the OD_260_ group and values with different lowercase letters were significantly different (*p* < 0.05) in the OD_280_ group **(G)**. The error bars represent standard deviations of the mean (n = 3).

As shown in Figure 4A, the OD_600_ of *P. bifermentans spores* treated with licorice extract (≤ 0.39 mg/ml) was significantly (*p* < 0.05) higher than that of other groups after 12 h of incubation, indicating that the 0.39 mg/ml licorice extract was sufficient to block the outgrowth of *P. bifermentans* spores. Therefore, the outgrowth curve of *P. bifermentans* spores was plotted based on MIOC (Figures 4B,C). Within 6 h of inoculation, few increases of OD_600_ were found as a control group in the absence of licorice extract, thereafter, a rapid increase emerged. The same phenomenon was found in the treatment group added with 1/2 × MIOC (0.19 mg/ml). However, when treated with licorice extract (1 × MIOC, 0.39 mg/ml and 2 × MIOC, 0.78 mg/ml), the increase of OD_600_ was obviously suppressed throughout the incubation period. Collectively, the MIOC value of *P. bifermentans* spores was 0.39 mg/ml, which is less than the concentration required to inhibit germination. Alanazi et al. (2018) investigated that the same concentrations of cinnamaldehyde, eugenol, allylisothiocyanate, and carvacrol showed higher inhibitory activity in the outgrowth of *C. perfringens* spores than in germination. In another study, Alnoman et al. (2015) demonstrated that the concentrations of sorbate and benzoate required against the germination of *C. perfringens* were relatively higher than the outgrowth. Hence, the outgrowth of spores could be the susceptible stage for various inhibitors. However, compared to outgrowth, chitosan exhibited a higher extent of inhibition on *C. perfringens* spore germination (Alnoman et al., 2017), which might be contributed to the higher susceptibility of the germinating spores to chitosan than outgrowing spores. The main antimicrobial mechanism of chitosan involves the binding of positively charged groups to negatively charged groups. Chitosan might lose some antimicrobial characteristics due to the competitive interaction of the cations in the existing medium with negatively charged bacterial cell walls (Kong et al., 2010).

The inhibition effect of the five bioactive ingredients identified by LC-MS on the outgrowth of spores was also determined. The outgrowth of *P. bifermentans* spores could be completely restrained at concentrations of 0.016 when dealt with glycyrrhizic acid and liquiritin apioside. The same phenomenon occurred when liquiritin was at least 0.031 mg/ml. MIOC of Licoisoflavone A and Liquiritigenin was 0.63 mg/ml (Table 3). The difference in their inhibitory effect may also be related to the content of hydroxyl. The hydroxyl group of some compounds in licorice can interact with the spore membranes by hydrogen bonding, leading to the destruction of membranes (Cai et al., 2019). Thus, compounds containing more hydroxyl groups showed better inhibitory activity, which gives an explanation for the better inhibitory effect of glycyrrhizic acid and liquiritin apioside.

**Table 3:** Inhibitory effect of bioactive compounds in licorice extract on the outgrowth of *P. bifermentans* spores.

**Germinant**	**Measurement index**	**Bioactive compound**				
		**Glycyrrhizic acid**	**Liquiritin**	**Liquiritin apioside**	**Licoisoflavone A**	**Liquiritigenin**
co-AI	MIOC	0.016	0.031	0.016	0.063	0.063
DPA	(mg/ml)	0.016	0.031	0.016	0.063	0.063

Outgrowing spores induced by the combination-nutrient-germinant (150 mmol/L L-alanine and 20 mmol/L inosine, co-AI) and non-nutrient-germinant (8 mmol/L dipicolinic acid, DPA) were incubated anaerobically in reinforced clostridium medium (RCM) containing licorice extract at 37°C for 12 h to obtain the minimum inhibitory spore outgrowth concentration (MIOC) of each compound. Results are averages of three trials and expressed as the means ± standard error.

**Figure 5:**
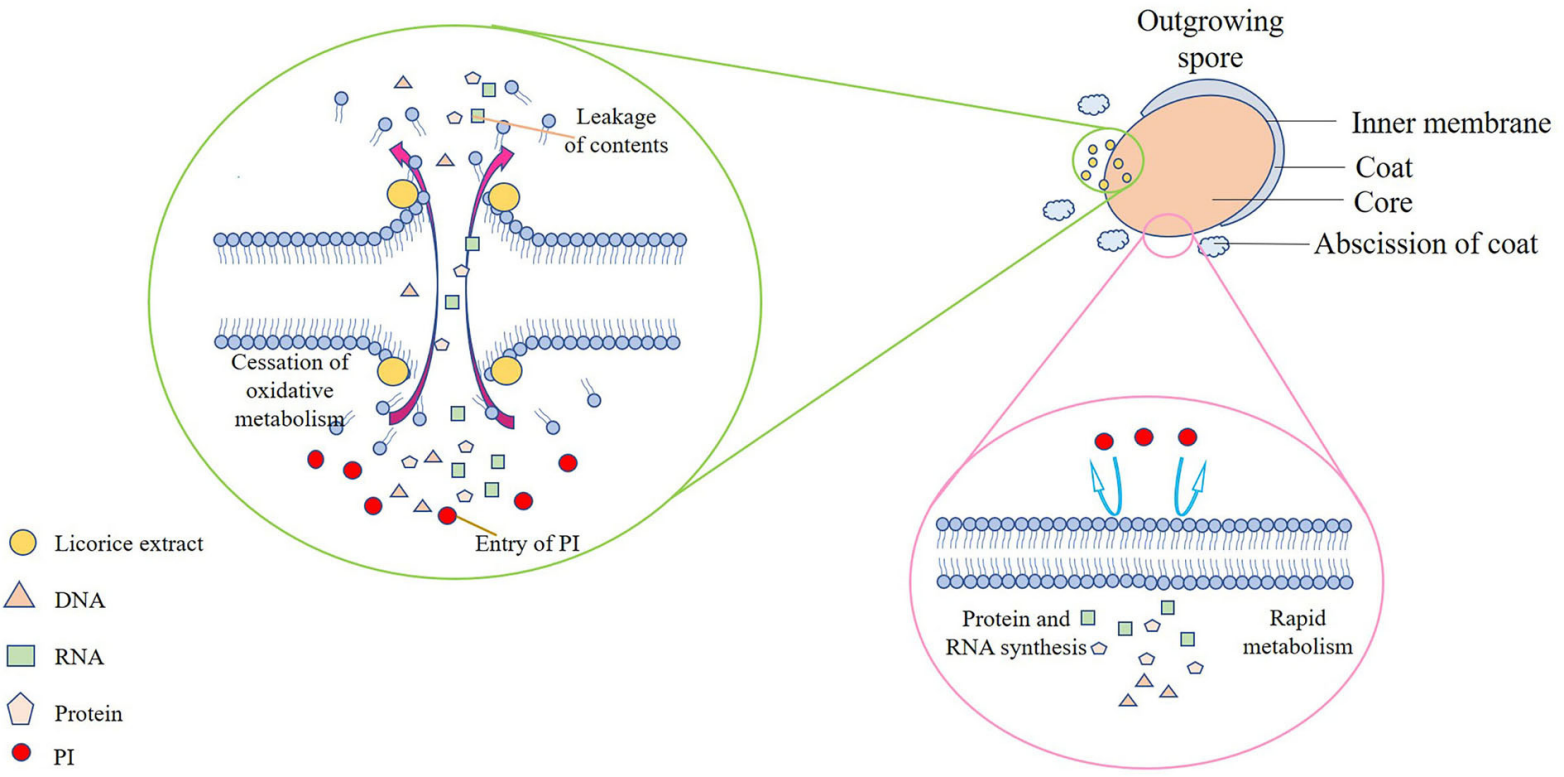
Schematic model of the *P. bifermentans* spore outgrowth inhibited by licorice extract. The spore inner membranes are more accessible for licorice extract due to the hydrolysis of the cortex and the abscission of the coat, causing the leakage of contents, the cessation of oxidative metabolism, and the entry of PI. On the contrary, no PI can enter the complete membranes, and metabolism and synthesis proceeded normally when licorice extract is absent.

Therefore, the mechanism of the outgrowth of *P. bifermentans* spore inhibited by licorice extract was investigated. Contrary to germination-inhibited spores, when outgrowing spores were exposed to licorice extract (MIOC, 0.39 mg/ml) and cultivated for 3 h, red fluorescence emitted by the spores was abundant as shown in Figures 4D_2_,D_6_. The population of spores with red fluorescence was further increased when the outgrowing spores were maintained in licorice solution (MIOC, 0.39 mg/ml) for 6 h, exhibiting that the extension of treatment time aggravated the damage to the outgrowing spores (Figures 4D_4_,D_8_). However, no red fluorescence was observed in the outgrowing spores of control, regardless of the cultivation duration (Figures 4D_1_,D_3_,D_5_,D_7_). Exposure of the outgrowing spores to licorice solution (MIOC) significantly (*p* < 0.05) reduced the viable count, and the increase in treatment time had an additive inactivation effect on the spores. The survival rate of outgrowing spores dropped to nearly 65% when retained in licorice solution (MIOC, 0.39 mg/ml) for 6 h (Figure 4E). It has been proven that the non-germinated spores are metabolically inactive (Setlow et al., 2001). Therefore, the oxidative metabolism of spores in the outgrowth stage was measured. As shown in Figure 4F, the increased content of formazan was found in the absence of licorice extract after 3 h of incubation, and a massive generation of formazan was detected from 6 to 12 h. On the contrary, the production of formazan remained at a low but detectable level during the whole stage of outgrowth with the addition of licorice extract, indicating the reduction of tetrazolium to formazan caused by oxidative metabolism was inhibited. A similar blockade of oxidative metabolism has been found when the membrane of *Bacillus* spores was destroyed by nisin (Gut et al., 2008). In addition, previous studies explained that the cessation of oxidative metabolism of spores could be related to a loss of membrane integrity (Sunny-Roberts and Knorr, 2008). In the presence of licorice extract, leakage of nucleic acid and protein significantly (*p* < 0.05) increased with extending exposure time and the differences between the treatment and control groups became increasingly significant (*p* < 0.05) (Figure 4G). The increased leakage of contents may be due to the more severe membrane breakage. Therefore, it could be estimated that the inhibitory effect of licorice extract on the outgrowth of *P. bifermentans* spores might be due to the loss of membrane integrity. Due to the abscission of the outer spore structures, the outgrowing spores no longer have tolerance. Therefore, the spore inner membranes are more accessible for licorice extract. The absence of oxidative metabolism also appeared because of membrane damage, resultant protein and RNA cannot be synthesized. Furthermore, the leakage of DNA, RNA, and protein aggravated the extent of damage, which undoubtedly broke the way for spores to outgrow. Therefore, the licorice-mediated membrane breakage is essential to prevent spores from being vegetative cells (Figure 5).

### Mechanism of the germination and outgrowth of *P. bifermentans* Spore inhibited by licorice extract

**Figure 6:**
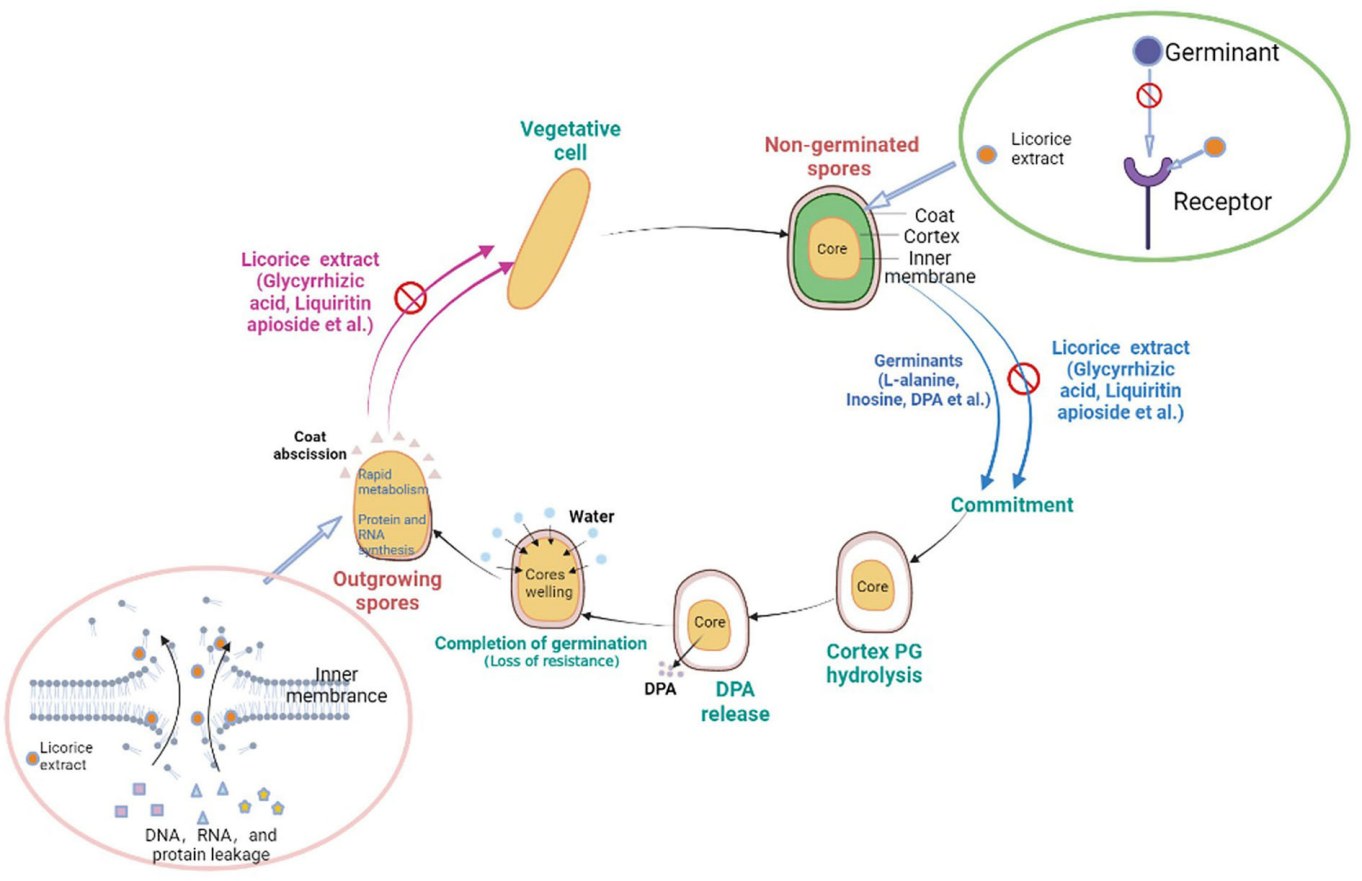
Schematic model for licorice extract to inhibit the germination and outgrowth of *P. bifermentans* spores. The non-germinated spores will be triggered by germinants such as L-alanine, inosine, and dipicolinic acid (DPA). On the one hand, prior to germination, the addition of licorice extract (glycyrrhizic acid, liquiritin apioside, etc.) will prevent the binding of germinants to receptors, resulting in the no occurrence of germinant sensing. On the other hand, the outgrowing spores, upon the hydrolysis of the cortex, the release of DPA, and the abscission of the coat, lose their resistance. Treatment with licorice extract at this point will cause damage to the inner membrane of the outgrowing spores and prevent them from becoming vegetative cells.

Licorice extracts can control *P. bifermentans* spores by directly inhibiting germination or destroying outgrowing spores (Figure 6). The coat and cortex provide protection to spores, which increases the difficulty for licorice extract to directly destroy the non-germinated spores. The inhibitory effect of licorice extract may be attributed to blocking the binding of the germinants and receptors. The cortex hydrolysis of *P. bifermentans* spores occurs at early germination (Setlow et al., 2017), therefore, the protective system for the spores gradually disintegrated. Subsequently, the release of DPA led to a further disappearance of resistance of spores and the abscission of the coat represents the loss of the last protective sheath of the spore, providing access to the spores for licorice extract that can lead to the destruction of membrane. The suspension of oxidative metabolism, the leakage of contents, and the disappearance of membrane integrity cause spore outgrowth interruption, obstructing cell wall biosynthesis and preventing the outgrowing spores from becoming vegetative cells (Gut et al., 2011). The information on the anti-spore mechanism of bioactive substances in the licorice extract is insufficient and future studies could focus on their structure-inhibition effect relationship.

## Conclusion

This study demonstrated that the licorice extract inhibited the germination and outgrowth of *P. bifermentans* spores. Licorice extract may inhibit germination by blocking the germinant sensing since the no damage to the structure of spores caused by licorice extract. In addition, with the treatment of licorice extract, the inner membrane of outgrowing spores was broken, causing the outgrowing spores to fail to become vegetative cells. Glycyrrhizic acid and liquiritin apioside played important roles in inhibiting the germination and outgrowth of *P. bifermentans* spores. Therefore, this study suggests that licorice extract can be used as a promising inhibitor to control spores.

## Data availability statement

The original contributions presented in the study are publicly available. This data can be found here: https://www.ncbi.nlm.nih.gov/nuccore/ON078500.1/.

## Author contributions

MS, YL, and MH contributed to the conception and design of the study. YX and KS contributed to the data analysis. MS wrote the first draft of the manuscript. YL and AA were involved in the revision of the manuscript. TH and JH performed the laboratory work. All authors contributed to the article and approved the final draft.

## Funding

This study was supported by the Agriculture Research System of China (CARS-41-Z), Jiangsu Provincial Agricultural Science and Technology Independent Innovation Fund (CX (22) 3194), and Taizhou Science and Technology Support Plan (Agriculture) Project (TN202112).

## Conflict of interest

Author TH was employed by Nanjing Huangjiaoshou Food Science and Technology Co., Ltd. The remaining authors declare that the research was conducted in the absence of any commercial or financial relationships that could be construed as a potential conflict of interest.

## Publisher's note

All claims expressed in this article are solely those of the authors and do not necessarily represent those of their affiliated organizations, or those of the publisher, the editors and the reviewers. Any product that may be evaluated in this article, or claim that may be made by its manufacturer, is not guaranteed or endorsed by the publisher.
