# Supplementary material for: Inhibitory effect of licorice extract on the germination and outgrowth of Paraclostridium bifermentans spores
Source: Front Microbiol. 2022 Dec 2;13:1076144. doi: 10.3389/fmicb.2022.1076144 (PMC9755857; doi:10.3389/fmicb.2022.1076144)
Supplement: Supplementary file 1 [file Data_Sheet_1.docx]

1. *Preparation of* *germinant solutions*

Germinants used in this study were: D-glucose, D-fructose, D-galactose, D-xylose, L-alanine, L-aspartate, L-valine, L-proline, L-histidine, L-leucine, inosine, dipicolinic acid (DPA). They were purchased from Haibo Biotechnology Co., Ltd. (Qingdao, China). Germinant solutions were prepared in 25 mmol/L Tris-HCl buffer (pH = 7.4), and adjusted to suitable concentrations, then filter sterilized (0.45 µm pore size), stored at 4 °C.

1. *Determination of germination rate*

*P. bifermentans* spore suspensions were heat-activated at 80 °C for 15 min and cooled in cold water for 5 min. Then the spore suspension was mixed with an equal amount of the germinant solutions and incubated anaerobically at 37 ℃ for 1 h. Determination of absorbance values at 600 nm for the treated spores. The germination rate (%) was calculated as follows (Eq. (1)):

$Germination rate \left( \% \right)={A_{T}}/{A_{0}}\times100\%$ (1)

A*_T_*: decrease of OD_600_; A_0_: initial OD_600_ of spores.

*3. Further verification of spore germination*

The germinated spores were killed by heat treatment since the resistance of spores would decrease after germination, while the non-germinated spores survived. Therefore, the thermal inactivation of *P. bifermentans* spores induced by germinant solutions was measured. Induced spores were exposed to a water bath at 80 ℃ for 20 min, then immediately immersed into a cold-water bath. Subsequently, 100 μL diluents with appropriate concentrations were inoculated onto reinforced clostridium agar (RCA) under anaerobic conditions at 37 ℃ for 24 h, and the survivor enumerations were conducted.

.


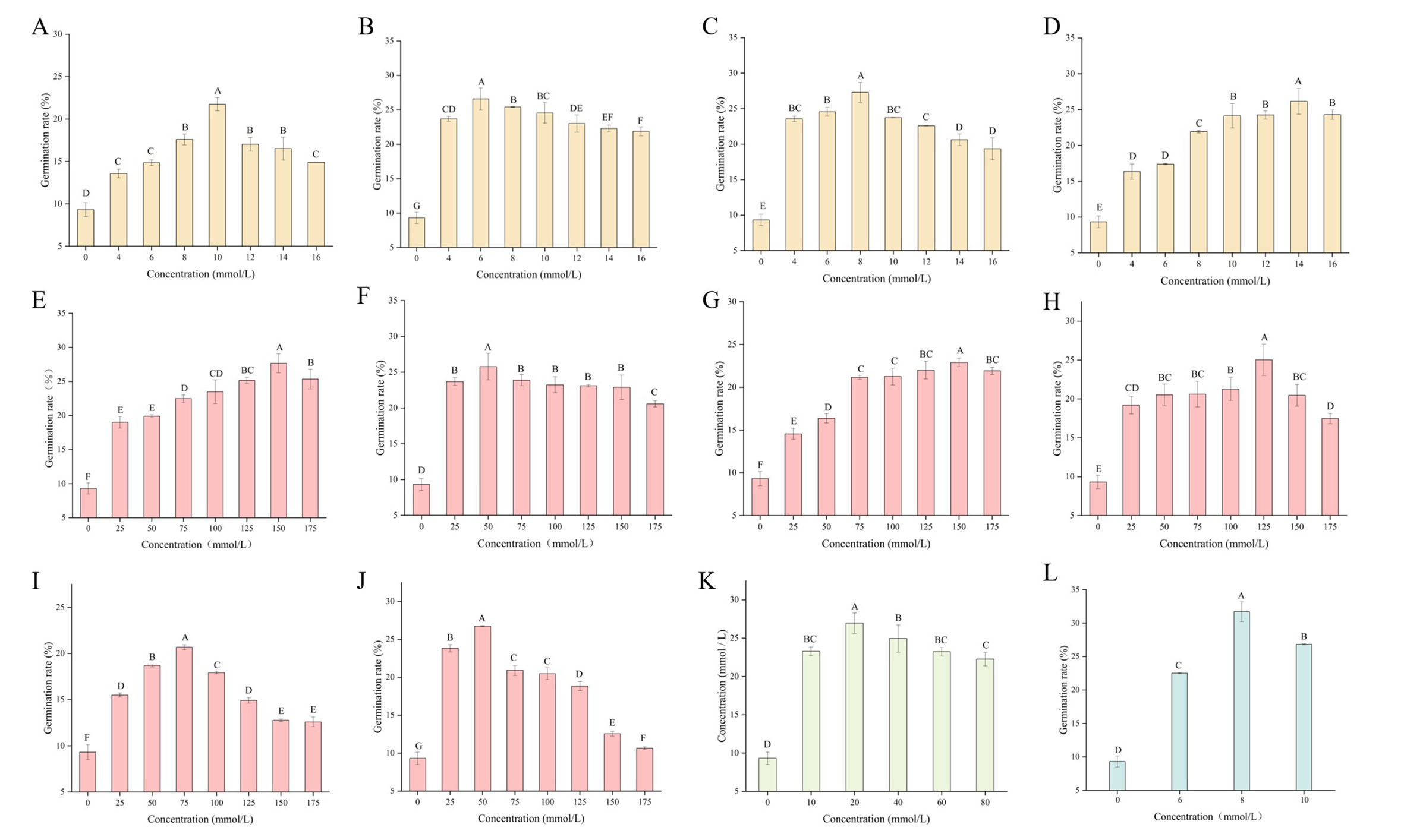


**Figure1.** Effects of different single germinants on the germination rate of *P. bifermentans* spores. **(A)** D-glucose, **(B)** D-fructose, **(C)** D-galactose and **(D)** D-xylose, the concentrations of the four D-sugars were adjusted to 0, 4, 6, 8, 10, 12, 14, 16 mmol/L; **(E)** L-alanine, **(F)** L-aspartate, **(G)** L-proline, **(H)** L-valine, **(I)** L-histidine and **(J)** L-leucine, the concentrations of the seven L-amino acids were adjusted to 0, 25, 50, 75, 100, 125, 150, 175 mmol/L; **(K)** inosine, the concentrations were adjusted to 0, 10, 20, 40, 60, 80 mmol/L; **(L)** dipicolinic acid (DPA), the concentrations were adjusted to 0, 6, 8, 10 mmol/L. Error bars represent standard deviations of the mean (n = 3). Values with different uppercase letters were significantly different (*P <* 0.05) in different concentrations.

**Table 1.** *P. bifermentans* spore germination in the presence of single germinants

| Germinants | Germination rate (%) | | | Thermal inactivation  -log (N_t_/N_0_) | |
| --- | --- | --- | --- | --- | --- |
| D-glucose  (10 mmol/L) | | 21.77±1.11^C^ | 1.31±0.02^H^ | |  |
| D-fructose  (6 mmol/L) | | 26.57±1.56^ABC^ | 1.76±0.01^D^ | |  |
| D-galactose  (8 mmol/L) | | 27.29±1.92^AB^ | 1.95±0.05^B^ | |  |
| D-xylose  (14 mmol/L) | | 26.09±2.08^BC^ | 1.76±0.01^D^ | |  |
| L-alanine  (150 mmol/L) | | 27.77±2.55^AB^ | 1.99±0.09^B^ | |  |
| L-aspartate  (50 mmol/L) | | 25.66±2.02^BC^ | 1.61±0.02^E^ | |  |
| L-proline  (125 mmol/L) | | 25.04±2.07^BC^ | 1.52±0.05^F^ | |  |
| L-valine  (150 mmol/L) | | 22.83±2.07^BC^ | 1.39±0.01^G^ | |  |
| L-leucine  (50 mmol/L) | | 26.72±0.57^ABC^ | 1.84±0.02^C^ | |  |
| Inosine  (20 mmol/L) | | 26.92±0.10^ABC^ | 1.79±0.03^CD^ | |  |
| Dipicolinic acid (DPA)  (8 mmol/L) | | 31.79±2.94 ^A^ | 3.06±0.01^A^ | |  |
| Control | | 9.66±0.85^D^ | 0.53±0.05^I^ | |  |

Single germinants with optimum concentration were selected and their ability to promote germination was further determined. N_t_: survivors after being treated in a water bath at 80 ℃ for 20 min; N_0_: initial number of spores. Results are averages of 3 trials and expressed as the means ± standard error. Different letters on the same column indicate significant differences (*P <* 0.05) between different groups.

**Table 2.** *P. bifermentans* spore germination in the presence of compound germinants

| Germinants | | Germination rate (%) | | Thermal inactivation  -log (N_t_/N_0_) |
| --- | --- | --- | --- | --- |
| D-fructose and L-alanine | 20.60±1.83^C^ | | 1.35±0.02^E^ | |
| D-fructose and L-leucine | 23.08±2.04^BC^ | | 1.40±0.14^E^ | |
| D-fructose and Inosine | 26.90±1.67^AB^ | | 1.84±0.06^B^ | |
| D-galactose and L-alanine | 25.88±1.79^AB^ | | 1.76±0.02^BC^ | |
| D-galactose and L-leucine | 25.88±1.18^AB^ | | 1.78±0.02^BC^ | |
| D-galactose and Inosine | 23.35±1.59^BC^ | | 1.58±0.08^D^ | |
| L-alanine and Inosine | 30.13±0.91^A^ | | 2.95±0.25^A^ | |
| L-leucine and Inosine | 23.53±1.06^BC^ | | 1.45±0.03^E^ | |
| D-fructose, L-alanine, and Inosine | 24.66±1.75^BC^ | | 1.67±0.09^CD^ | |
| D-fructose, L-leucine, and Inosine | 26.53±0.83^AB^ | | 1.83±0.03^B^ | |
| D-galactose, L-alanine, and Inosine | 25.77±0.42^AB^ | | 1.72±0.02^BC^ | |
| D-galactose, L-leucine, and Inosine | 25.60±2.03^AB^ | | 1.73±0.02^BC^ | |
| D-fructose, D-galactose, L-alanine, L-leucine, and Inosine | 24.35±0.80^BC^ | | 1.66±0.06^CD^ | |
| Control | 10.96±0.40^D^ | | 0.54±0.03^F^ | |

D-fructose (10 mmol/L), D-galactose (8 mmol/L), L-alanine (150 mmol/L), L-leucine (50 mmol/L), and inosine (20 mmol/L) were combined and their ability to promote germination was further determined. N_t_: survivors after being treated in a water bath at 80 ℃ for 20 min; N_0_: initial number of spores. Results are averages of 3 trials and expressed as the means ± standard error. Different letters on the same column indicate significant differences (*P <* 0.05) between different treatment groups.


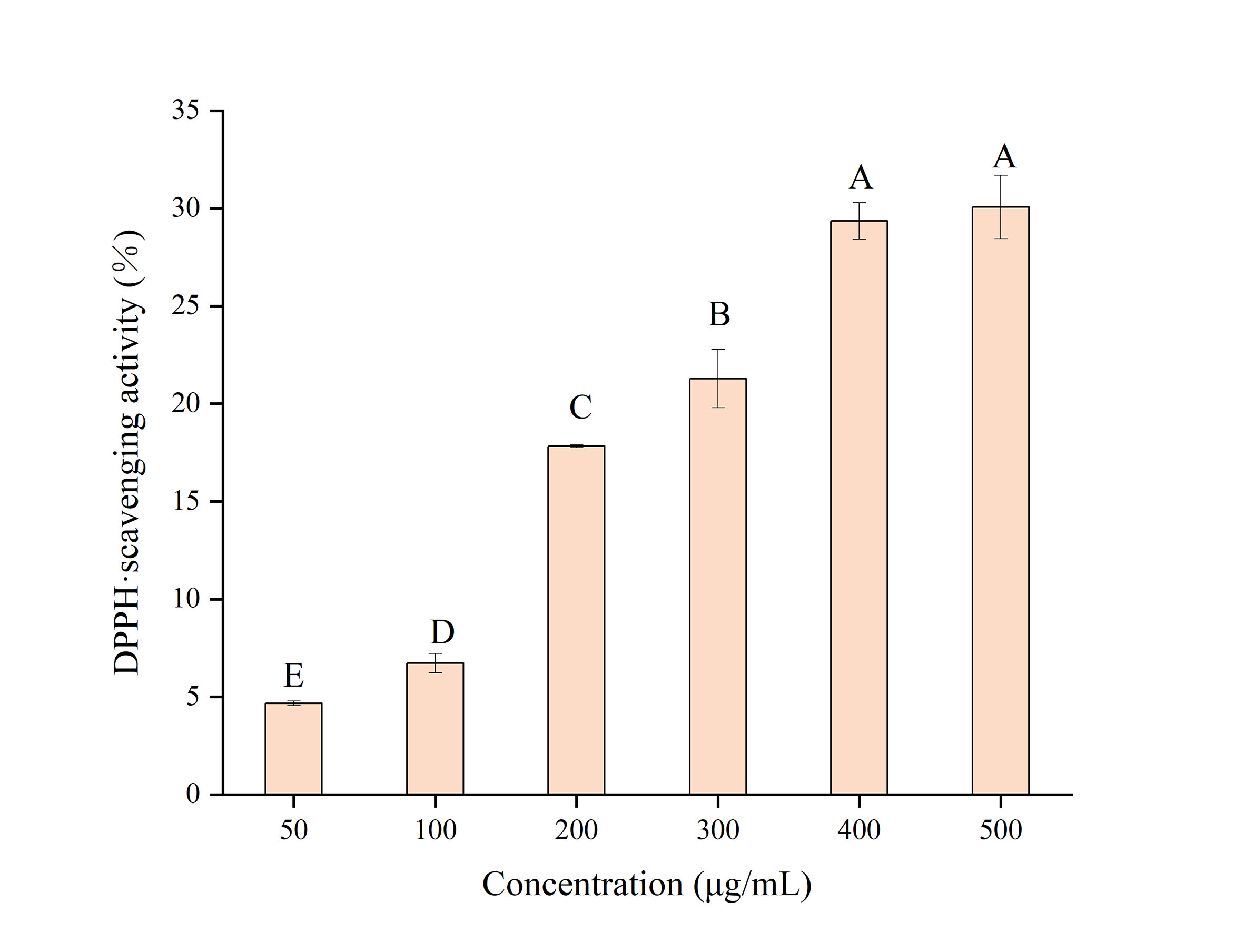


**Figure 2.** DPPH radical dot clearance rate of licorice extract with different concentrations. Values with different uppercase letters, within different concentrations, were significantly different (*P <* 0.05). Error bars represent standard deviations of the mean (n = 3).
